# Hypochondriasis, somatic amplification, and pain beliefs in patients with medication overuse headache

**DOI:** 10.1055/s-0046-1824430

**Published:** 2026-07-03

**Authors:** Zeynep Ziroglu, Burçin Çolak, Hafize Nalan Güneş, Şeval Ozaydınlı, Levent Ertuğrul İnan

**Affiliations:** 1 Health Sciences University, Ankara Training and Research Hospital, Department of Neurology, Ankara, Turkey.; 2 Ankara University, Faculty of Medicine, Department of Psychiatry, Ankara, Turkey.; 3 Ankara University, Brain Research Center, Ankara, Turkey.

**Keywords:** Headache Disorders, Secondary, Hypochondriasis, Somatization Disorder

## Abstract

**Background:**

Medication overuse headache (MOH) is a chronic headache (CH) that develops or worsens due to medication overuse. Somatization and hypochondriasis, as well as beliefs about the experienced pain, may accompany many chronic pain situations from a holistic perspective.

**Objective:**

This study aimed to compare MOH patients with CH and episodic headache (EH) in terms of somatization, hypochondriasis, and beliefs and attitudes on pain.

**Methods:**

The Hospital Depression and Anxiety Scale (HADS), the Somatosensory Amplification Scale (SSAS), the Pain Beliefs Questionnaire (PBQ), the Pain Catastrophizing Scale (PCS), Eysenck Personality Questionnaire Revised–Short Form (EPQR-S), and Whiteley index-7 (WI-7) were administered to the participants with MOH (n = 80), CH (n = 81), and EH (n = 79).

**Results:**

There were no group differences in terms of somatic amplification between MOH and CH group. A high WI_Total score (lower hypochondriacal tendency) was predictive in separating the MOH group from the CH one. The score obtained from the PBQ_Organic subscale in the MOH group was significantly lower than the EH group, while the PCS_Total, PCS_Helplessness, PCS_Rumination, and HADS scores were significantly higher ( *p* < 0.05).

**Conclusion:**

Our results indicate that the patients in the MOH group had more organic pain beliefs, felt more helpless in case of pain, and experienced exaggeration and rumination more than the EH group, while hypochondriasis was lower than in the CH group. This could indicate that patients with MOH do not seek further treatment that can lead to more headache medication use without medical examination.

## INTRODUCTION

The worldwide prevalence of chronic daily headache is 3 to 5%. 1 Medication-overuse headache (MOH) is an important public health problem, reported in 30 to 50% of chronic headache (CH) patients seen in specialized clinics and tertiary centers, although its general population prevalence is 1 to 1.7%. 2 3 4 5 6 According to the International Classification of Headache Disorders 3rd edition (ICHD-3) criteria, MOH is defined as headaches on ≥ 15 days/month due to acute/symptomatic medication use for more than 3 months, with medication intake exceeding 10 to 15 days/month depending on the drug subtype. 7 Headache typically develops or worsens with overuse and improves within 2 months after discontinuation. 7 Although most common in migraine and tension-type headaches (TTH), MOH may occur with all subtypes. 7 8

Previous studies have demonstrated associations between migraine or TTH and various psychiatric comorbidities. 9 10 While some researchers suggest that similar comorbidities may contribute to the transformation from EH to MOH, it remains unclear whether these are risk factors or consequences of MOH. 11

Studies examining personality traits in MOH have reported potential links with hypochondriasis and somatization. 12 13 Mood disorders, anxiety disorders, obsessive–compulsive traits, and personality disorders have been found to be more common in MOH than in episodic headaches (EH) or CH. 13 14 15 16 Psychiatric comorbidity is also frequent in individuals with TTH who develop MOH, similar to those with migraine. 17 According to the Diagnostic and Statistical Manual of Mental Disorders, 4th edition (DSM-IV) criteria, 2/3 of MOH patients meet substance dependence criteria, and high substance dependence scale (SDS) scores have been reported. 18 19 Some authors propose that psychiatric comorbidity may reflect low pain tolerance, and that in the opiate overuse subgroup, medications may be used both for pain relief and to cope with psychological stress. 17

Cognitive factors may also influence pain perception and coping. Pain beliefs, shaped by psychological and social factors, affect how patients perceive and physicians manage pain. The Pain Beliefs Questionnaire (PBQ) includes organic and psychological subscales evaluating beliefs about the causes and treatment of pain. 20 Pain catastrophizing, assessed by rumination, magnification, and helplessness subscales, reflects exaggerated negative thoughts about pain and is associated with pain severity and disability. 21 Our clinical hypothesis was that patients with MOH would exhibit greater hypochondriasis tendencies and symptom exaggeration compared with control groups, and that cognitive characteristics such as pain beliefs and pain catastrophizing might further differentiate MOH patients from others.

## METHODS

### Participants

This case–control study was conducted in the neurology outpatient clinic of a tertiary hospital in Turkey between January and March 2023, in accordance with the Declaration of Helsinki (1975, revised in 2000) and approved by the Health Sciences University Non-Interventional Research Ethics Committee in November 22, 2023, number E-93471371-514.99-229973905.

All adult patients who consecutively presented to the outpatient clinic during the study period were screened for eligibility. Patients meeting the inclusion and exclusion criteria were enrolled without prior selection or randomization and were assigned to study groups based on their clinical diagnosis at presentation.

A total of 80 patients with drug-overuse headache, 81 with chronic TTH or migraine without overuse, and 79 with episodic TTH or migraine were included. The exclusion criteria were: age < 18 years, secondary headache, primary headaches other than migraine and TTH, and use of prophylactic treatment.

### Sample size calculation

The a-priori sample size calculation was performed using G*Power software (Heinrich Heine University), version 3.1.9.7. Based on a one-way analysis of variance (ANOVA) with three independent groups, an effect size of Cohen's f = 0.27, a two-sided significance level of α = 0.05, and a desired statistical power of 95%, the minimum required total sample size was calculated as 216 participants (72 per group). Accordingly, we planned to enroll approximately 80 participants in each group to compensate for potential exclusions and missing data. Due to incomplete questionnaires and exclusion after eligibility reassessment, the final analyzed sample consisted of 80 patients with medication-overuse headache, 81 with CH, and 79 with EH. The assumed effect size (Cohen's f = 0.27) was determined based on previously published studies reporting differences in personality traits among episodic and chronic migraine patients.

### Procedure

All patients underwent detailed history-taking and neurological examination. Diagnoses were made by an experienced neurologist according to ICHD-3 criteria. 22 Group allocation was based solely on these diagnostic criteria. Written informed consent was obtained. Clinicians completed the sociodemographic form, while participants completed the self-report scales after their outpatient evaluation; personnel assisted when clarification was needed.

### Data collection tools

#### *Sociodemographic data*

Information was obtained about the participants' age, gender, education level, socioeconomic status, marital status and history of being cheated.

#### *Headache data collection*

Duration-frequency-severity of headache, symptomatic medications used for headache and for MOH group patients which medications were over-used, duration of over-use and number of over-used medications per month were recorded.

#### *Hospital Depression and Anxiety Scale (HADS)*

The Hospital Depression and Anxiety Scale (HADS), with a validated and reliable Turkish version, was designed to measure anxiety and depression in clinical groups. 23 24 High scores are associated with higher levels of both psychological conditions.

#### *Somatosensory Amplification Scale* ( *SSAS)*

The Somatosensory Amplification Scale (SSAS) assesses the tendency to perceive somatic and visceral sensations as unusually intense or distressing. 25 Its Turkish validity and reliability were established by Güleç and Sayar in 2007. 26 The scores range from 10 to 50, with higher ones indicating greater somatization.

#### *Pain Beliefs Questionnaire (PBQ)*

The Pain Beliefs Questionnaire (PBQ) consists of two subscales (organic and psychological beliefs) about the causes and treatment of pain. It is validated in Turkish, and reliability has been proven. 20 27 Three scores are measured for both subscales and for the total test. A lower score indicates that the pain beliefs related to that test are higher.

#### *Pain Catastrophizing Scale (PCS)*

The Pain Catastrophizsing Scale (PCS) consists of rumination, magnification and helplessness subscales used to measure exaggerated negative thoughts and feelings about pain, and a Turkish validity and reliability study was also conducted. 21 28 Increasing scores are correlated with increasing pathology.

#### *Eysenck Personality Questionnaire Revised–Short Form (EPQR-S)*

The Eysenck Personality Questionnaire Revised–Short Form (EPQR-S) has already been validated in Turkish. It assesses extraversion, neuroticism, and psychoticism. 29 30 31 In this study, only the Neuroticism subscale was used, as previous research indicates that neuroticism is more common in patients with chronic migraine and TTH. 32 33 34

#### *Whiteley Index-7*

The Whiteley Index-7 (WI-7) is a self-report measure for screening hypochondriasis, consisting of two subscales: somatic symptoms/somatic preoccupation and disease anxiety/phobia. It has a validated Turkish version. 35 36 37 Lower scores indicate greater hypochondriacal tendency.

### Statistical analysis

The IBM SPSS Statistics for Windows (IBM Corp.) software, version 20.0, was used in the study. Categorical variables were expressed as frequencies (n) and percentages (%). Continuous variables were expressed as arithmetic means, standard deviations (SDs), and median values by testing their conformity to a normal distribution with the Skewness and Kurtosis measures.

For comparison of the scales between the groups, ANOVA and post hoc Bonferroni correction procedures were used. Pearson's correlation analysis was used for the continuous variables. Categorical variables were analyzed with Pearson's Chi-squared (χ ^2^ ). To estimate whether the scales applied differentiated the MOH, CH, and EH groups from one another, prior to multivariable regression analysis, collinearity diagnostics were performed to assess potential multicollinearity among the candidate predictor variables.

Age was included as a covariate due to its established association with headache chronicity and the observed between-group differences. Preliminary analyses demonstrated that age was strongly associated with education level, occupational status, and marital status. Therefore, to avoid redundancy and overfitting, age was retained as a parsimonious covariate representing sociodemographic characteristics. Findings were given at a *p* < 0.05 significance level and 95%CI.

## RESULTS

### Sociodemographic characteristics of the participants

The study included 240 participants (85% female, n = 204; 15% male, n = 39) with a mean age of 35.07 ± 12.67 (range: 18–78) years. The MOH group was significantly older than the EH group (F[2] = 4.824, *p* = 0.009). Gender and socioeconomic level did not differ between groups (χ ^2^ [2] = 4.399, *p* = 0.111; χ ^2^ [2] = 0.598, *p* = 0.463).

Both MOH and CH participants had lower education levels and were more likely to be married and unemployed compared to EH (χ ^2^ [2] = 14.633, *p* = 0.006; χ ^2^ [2] = 8.771, *p* = 0.012; χ ^2^ [2] = 15.555, *p* = 0.004). The CH group had a significantly higher rate of being cheated (χ ^2^ [2] = 22.428, *p* < 0.001). Sociodemographic data are summarized in Table 1 .

**Table 1:** Sociodemographic characteristics and distribution of primary headache diagnoses of the groups

		Group			χ ^2^ /F	* p* -value
		MOH (n = 80): n (%)	CH (n = 81) : n (%)	EH (n = 79) : n (%)		
**Primary headache**	CM	60 (75)	42 (51.581)	−	16.65	<0.001
	CTTH	20 (25)	39 (48.148)	−		
	EM	−	−	35 (44.303)		
	ETTH	−	−	44 (55.696)		
** Mean age, years ^a^**		38.30 ± 12.852	34.7 ± 12.766	32.19 ± 11.756	4.824*	0.009
**Gender**	F	69 (86.2)	73 (90.1)	62 (78.5)	4.399	0.111
	M	11 (13.8)	8 (9.9)	17 (21.5)		
**Education**	Primary school	29 (36.3)	18 (22.2)	14 (17.7)	14.633	0.006
	Middle–high school	36 _(_ 45.0)	33 (40.7)	30 (38.0)		
	Higher education	15 (18.8)	30 (37.0)	35 (44.3)		
**Occupation**	Housewife	49 (61.3)	50 (61.7)	30 (38.0)	15.555	0.004
	Student	10 (12.5)	7 (8.6)	21 (26.6)		
	Working	21 (26.3)	24 (29.6)	28 (35.4)		
**Socioeconomic level**	Low	15 (18.8)	13 (16.0)	10 (12.7)	3.598	0.463
	Middle	52 (65.0)	47 (58.0)	54 (68.4)		
	Upper-middle	13 (16.3)	21 (25.9)	15 (19.0)		
**Marriage status**	No	22 (27.8)	30 (37.0)	40 (50.6)	8.771	0.012
	Yes	57 (72.2)	51 (63.0)	39 (49.4)		
**Being cheated on**	No	68 (85.0)	54 (66.7)	75 (94.9)	22.428	<0.001
	Yes	12 (15.0)	27 (33.3)	4 (5.1)		

Abbreviations: χ ^2^ , Chi-squared; CH, chronic headache; CM, chronic migraine; CTTH, chronic tension-type headache; EH, episodic headache; EM, episodic migraine; ETTH, episodic tension-type headache; MOH, medication-overuse headache. Note: *Analysis of variance (ANOVA) test.

### Clinical characteristics of the MOH group

Chronic migraine was the accompanying primary headache in 75% of MOH patients. Mean headache duration was 102.5 ± 107.9 months, and mean VAS score (7.79 ± 1.65) was significantly higher than in CH and EH groups (MOH: *p* = 0.004; CH and EH: *p* < 0.001).

The MOH patients had a mean of 29.06 ± 38.7 months of medication overuse and used 29.06 ± 16.29 symptomatic drugs/month. Analgesics (83.75%) were the most commonly overused drugs, followed by ergotamine (7.5%), polyabusers (7.5%), and combined medications (1.25%). Clinical characteristics are presented in Table 2 .

**Table 2:** Clinical characteristics of MOH patients

MOH	(n = 80)
Mean age (years) ^a^	38.30 ± 12.852
Gender (F/M)	69/11
*Medication overuse (ICHD-II criteria subgroups): n (%)*	
Ergotamine	6 (7.5)
Triptans	0
Analgesics	67 (83.75)
Opioids	0
Combination medications	1 (1.25)
Polyabusers (more than one of the aforementioned categories)	6 (7.5)
Concomitant primary headache CM/CTTH	60/20
Mean duration of chronic headache (months)	102.5 ± 107.9
Mean headache VAS score	7.79 ± 1.65
Mean duration of symptomatic drug overuse (months)	29.06 ± 38.7
Mean doses taken (monthly)	29.06 ± 16.29

Abbreviations: CM, chronic migraine; CTTH, chronic tension-type headache; F, female; ICHD, International Classification of Headache Disorders; M, male; MOH, medication-overuse headache; VAS, visual analogue scale.

### Comparison of scale scores between groups

The MOH group had significantly lower PBQ_Organic scores than the EH group ( *p* = 0.013). PCS_Total, PCS_Helplessness, PCS_Rumination, and HADS_Depression were significantly higher in MOH and CH groups compared with EH (all *p* < 0.05).

The variable PCS_Magnification was higher in MOH versus EH ( *p* = 0.043). HADS_Total and HADS_Anxiety were also higher in MOH ( *p* = 0.016) compared with EH ( *p* = 0.049).

Other scales showed no significant group differences ( *p* > 0.05). Full scale comparisons are shown in Table 3 . For scales with reverse scoring (Whiteley index and pain beliefs questionnaire), lower scores indicate higher levels of health anxiety and maladaptive pain beliefs, respectively. All results are interpreted according to the original scoring directions of these instruments.

**Table 3:** Significant differences between cases and controls emerged from the analysis of variance and post hoc analysis

Dependent variable			MD (I-J)	SE	Sig.	95%CI	
						Lower bound	Upper bound
SSAS	MOH CH	CH	0.853	1.336	1.000	−2.37	4.07
		EH	2.720	1.344	0.132	−0.52	5.96
		EH	1.867	1.340	0.495	−1.36	5.10
EPQ_Neuroticism	MOH	CH	0.209	0.300	1.000	−0.52	0.93
		EH	0.659	0.302	0.090	−0.07	1.39
	CH	EH	0.451	0.301	0.408	−0.28	1.18
WI_SI	MOH	CH	0.124	0.257	1.000	−0.49	0.74
		EH	−0.354	0.258	0.513	−0.98	0.27
	CH	EH	−0.478	0.257	0.191	−1.10	0.14
WI_Hc	MOH	CH	0.145	0.093	0.361	−0.08	0.37
		EH	0.067	0.094	1.000	−0.16	0.29
	CH	EH	−0.078	0.093	1.000	−0.30	0.15
WI_Total	MOH	CH	0.265	0.304	1.000	−0.47	1.00
		EH	−0.291	0.306	1.000	−1.03	0.45
	CH	EH	−0.556	0.304	0.204	−1.29	0.18
PBQ_Organic	MOH	CH	−1.824	0.997	0.206	−4.23	0.58
		EH	‒2.889*	1.003	0.013*	−5.31	−0.47
	CH	EH	−1.065	1.000	0.863	−3.48	1.35
PBQ_Psychological	MOH	CH	−0.558	0.725	1.000	−2.31	1.19
		EH	−0.188	0.730	1.000	−1.95	1.57
	CH	EH	0.370	0.728	1.000	−1.38	2.12
PBQ_Total	MOH	CH	−2.382	1.444	0.301	−5.86	1.10
		EH	−3.077	1.453	0.106	−6.58	0.43
	CH	EH	−0.695	1.448	1.000	−4.19	2.80
PCS_Helplessness	MOH	CH	1.238	0.918	0.536	−0.97	3.45
		EH	4.338**	0.923	0.000**	2.11	6.56
	CH	EH	3.100**	0.921	0.003**	0.88	5.32
PCS_Magnification	MOH	CH	0.295	0.480	1.000	−0.86	1.45
		EH	1.191*	0.483	0.043*	0.03	2.35
	CH	EH	0.895	0.481	0.192	−0.27	2.06
PCS_Rumination	MOH	CH	0.545	0.785	1.000	−1.35	2.44
		EH	2.808**	0.790	0.001**	0.90	4.71
	CH	EH	2.263*	0.787	0.013*	0.36	4.16
PCS_Total	MOH	CH	2.079	2.000	0.899	−2.74	6.90
		EH	8.337**	2.013	0.000**	3.48	13.19
	CH	EH	6.259**	2.007	0.006**	1.42	11.10
HADS_Anxiety	MOH	CH	0.816	0.697	0.727	−0.86	2.50
		EH	1.695*	0.701	0.049*	0.00	3.38
	CH	EH	0.878	0.699	0.630	−0.81	2.56
HADS_Depresion	MOH	CH	0.025	0.543	1.000	−1.28	1.33
		EH	1.393*	0.546	0.034*	0.08	2.71
	CH	EH	1.368*	0.545	0.038*	0.05	2.68
HADS_Total	MOH	CH	0.841	1.092	1.000	−1.79	3.47
		EH	3.087*	1.099	0.016*	0.44	5.74
	CH	EH	2.246	1.096	0.124	−0.40	4.89

Abbreviations: CH, chronic headache; EH, episodic headache; EPQ, Eysenk personality questionnaire; HADS, hospital anxiety and depression scale; Hc, hypochondriasis; MOH, medication-overuse headache; PBQ, pain beliefs questionnaire; PCS, pain catastrophizing scale; SI, somatization illness; SSAS, somatosensory amplification scale; WI, Whiteley index. Notes: Values in bold indicate statistical significance.

* The mean difference is significant at the 0.05 level.

** The mean difference is significant at the 0.01 level.

### Correlation analysis of dependent variables

Pearson analysis revealed significant positive correlations between SSAS_Total, EPQR-S_Neuroticism, PCS subscales, and HADS scores ( *p* < 0.01).

**Figure 1:**
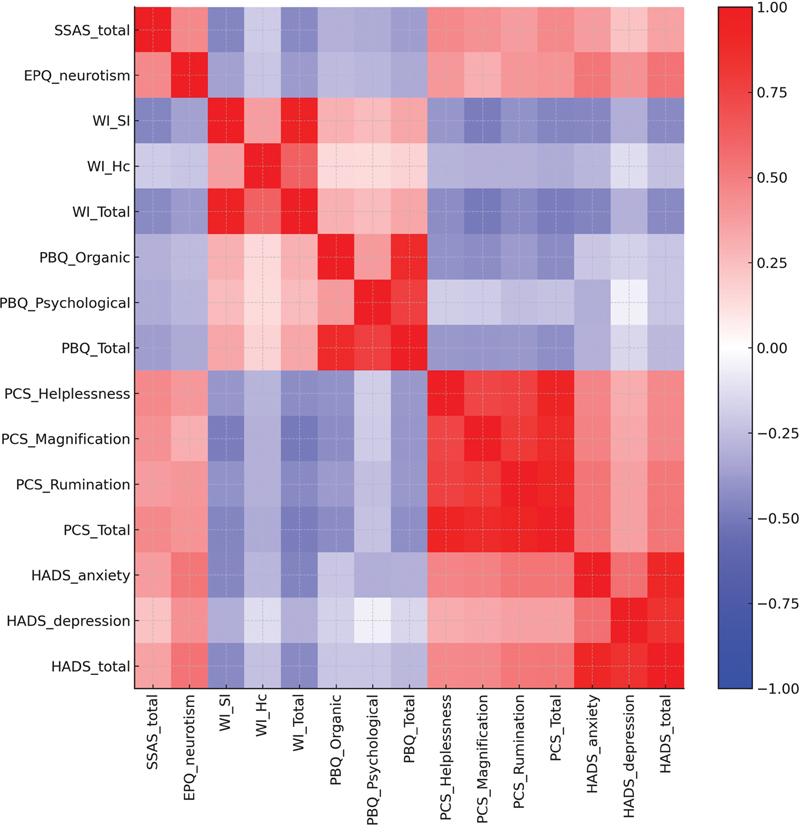
Abbreviations: SSAS, Somatosensory Amplification Scale; EPQ, Eysenk Personality Questionnaire; WI, Whiteley Index; SI, somatization illness; Hc, hypochondriasis; PBQ, Pain Beliefs Questionare; PCS, Pain Catastrophing Scale; HADS, Hospital Anxiety and Depression Scale. Correlation matrix heatmap showing Spearman correlation coefficients (ρ) between SSAS, EPQ, WI, PBQ, PCS, and HADS scores. Colors indicate correlation magnitude and direction (−1 to +1).

These variables were negatively correlated with WI-7_SI, WI-7_Hc, WI-7_Total, and PBQ subscales ( *p* < 0.01), which is consistent with the reverse scoring of these scales, where lower scores reflect higher levels of health anxiety and dysfunctional pain beliefs. The HADS_Depression showed no significant correlation with WI-7_SIorPBQ_Psychological ( *p* > 0.05) but was positively correlated with PBQ_Total ( *p* < 0.05). Significant positive correlations were also observed among WI and PBQ subscales themselves ( *p* < 0.05–0.01, Figure 1 ). Correlation results are summarized in **Supplementary Material** ( https://www.arquivosdeneuropsiquiatria.org/wp-content/uploads/2026/02/ANP-2025.0477-Supplementary-Material.docx ).

### Multinomial logistic regression for differentiating MOH, CH, and EH subgroups

Evaluation of multicollinearity demonstrated that none of the included variables exceeded acceptable collinearity thresholds, indicating that multicollinearity was not a concern for the regression analyses (SASS_total, Tolerance = 0.65, VIF = 1.53; EPQR-S_Neuroticism, Tolerance = 0.61, VIF = 1.65; WI-7_Total, Tolerance = 0.66, VIF = 1.52; PBQ_Total, Tolerance = 0.77, VIF = 1.31; PCS_Total, Tolerance **=** 0.57, VIF = 1.75; HADS_total, Tolerance = 0.57, VIF **=** 1.76).

**Figure 2:**
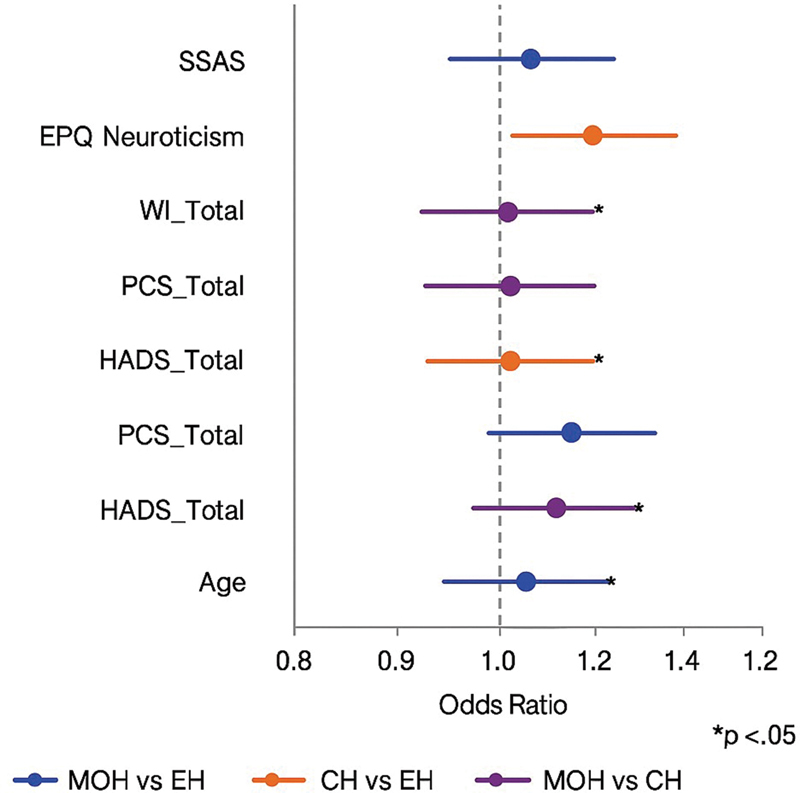
Abbreviations: MOH, medication-overuse headache; CH, chronic headache; EH, episodic headache; SSAS, Somatosensory Amplification Scale; EPQ, Eysenk Personality Questionare; WI, Whiteley Index; SI, somatization illness; Hc, hypochondriasis; PBQ, Pain Beliefs Questionare; PCS, Pain Catastrophing Scale; HADS, Hospital Anxiety and Depression Scale. Logistic regression model showing the effect of scale scores and age in predicting headache diagnostic groups.

Regression analysis showed that higher PCS_Total scores and older age predicted MOH versus EH. A higher WI-7_Total score predicted MOH versus CH. The PCS_Total also predicted CH versus EH model fit: R ^2^ = 0.129 (Cox–Snell), 0.145 (Nagelkerke); χ ^2^ (12) = 33.098, *p* = 0.003 ( Figure 2 ). Regression results are presented in Table 4 .

**Table 4:** Effect of the scores of the scales applied in identifying diagnostic groups

Group		B	SE	OR	95% CI	
MOH a	SSAS	0.018	0.025	1.018	0.970	1.069
	EPQ_Neuroticism	0.052	0.112	1.054	0.846	1.312
	WI_Total	0.159	0.112	1.173	0.942	1.460
	PBQ_Total	−0.006	0.021	0.994	0.953	1.036
	PCS_Total	0.044	0.018	1.045	1.009	1.081
	HADS_Total	0.028	0.032	1.029	0.966	1.096
	Age	0.037	0.014	1.038	1.009	1.067
CH a	SSAS	0.005	0.024	1.005	0.959	1.054
	EPQ_Neuroticism	0.016	0.108	1.016	0.823	1.255
	WI_Total	−0.051	0.105	0.950	0.773	1.167
	PBQ_Total	0.021	0.020	1.022	0.982	1.063
	PCS_Total	0.034	0.017	1.035	1.001	1.070
	HADS_Total	0.012	0.031	1.013	0.952	1.077
	Age	0.017	0.014	1.017	0.989	1.046
MOH b	SSAS	0.013	0.023	1.013	0.967	1.060
	EPQ_Neuroticism	0.036	0.110	1.037	0.835	1.287
	WI_Total	0.211	0.105	1.235	1.004	1.518
	PBQ_Total	−0.028	0.020	0.973	0.935	1.012
	PCS_Total	0.009	0.017	1.009	0.977	1.043
	HADS_Total	0.016	0.030	1.016	0.958	1.077
	Age	0.020	0.013	1.020	0.995	1.047

Abbreviations: CH, chronic headache; EH, episodic headache; EPQ, Eysenk personality questionnaire; HADS, hospital anxiety and depression scale; MD, mean difference; MOH, medication-overuse headache; PBQ, pain beliefs questionnaire; PCS, pain catastrophizing scale; SE, standard error; SSAS, somatosensory amplification scale; WI, Whiteley index.

Notes: * *p* < 0.05.

** *p* < 0.01; R ^2^ = 0.129 (Cox-Snell); 0.145 (Nagelkerke); Model Chi-squared (χ ^2^ ) (12) = 33.098; *p* = 0.003.

a The reference category is EH.

b The reference category is CH.

## DISCUSSION

In the biopsychosocial model suggested by Engel 38 instead of the traditional biomedical disease model, diseases are defined as a dynamic process that is mutually influenced by all biological, psychological, and social factors, and this is the dominant view in understanding chronic pain in current scientific societies. 38 39 Consistent with this perspective, previous studies have demonstrated strong associations between migraine, TTH, and psychiatric comorbidities. 34 Psychiatric symptoms have also been reported to be similar across patients with MOH, regardless of whether migraine or TTH is the underlying primary disorder, and may contribute to the transition from EH to MOH. 18 40 These findings provide the conceptual background for examining psychological and cognitive characteristics in MOH. In this context, Kristoffersen et al. demonstrated that brief educational interventions in primary care reduced headache frequency and medication overuse in MOH patients, without a parallel improvement in depression, anxiety, or disability, highlighting the need for a multidimensional treatment approach beyond pharmacological strategies. 15

Building on this literature, the present study focused on whether patients with MOH differ from CH and EH groups in terms of somatization, hypochondriasis, pain beliefs, and pain catastrophizing. To our knowledge, this is the first clinical study to specifically evaluate pain beliefs in patients with MOH, representing a novel contribution to the field.

Consistent with previous reports, higher depression and anxiety scores were observed in the MOH group compared with the EH group. 33 41 However, no significant differences were found between MOH and CH groups, supporting the notion that affective symptoms may be more closely related to headache frequency than to subtype. 11 Owing to the cross-sectional design, we cannot determine whether these psychiatric features predispose individuals to MOH or emerge as a consequence of CH. 40

When personality-related constructs were evaluated collectively using SSAS, EPQR-S, and WI-7 scores, no significant group differences were observed. This finding contrasts with some earlier reports suggesting greater hypochondriasis and somatization tendencies in MOH compared with EH or healthy controls. 12 13 Contrary to our expectations, multinomial regression analysis revealed that hypochondriasis was more prominent in the CH group than in the MOH group. This unexpected result may suggest that some patients with MOH represent a subgroup characterized by habitual analgesic use and reduced illness-focused concern, potentially leading to delayed healthcare-seeking behavior and persistent medication overuse.

Previous studies have suggested that psychiatric comorbidities in patients with medication-overuse headache may be associated with reduced pain tolerance, leading individuals to use analgesics not only for pain relief but also as a coping strategy for psychological distress, particularly in cases involving opioid overuse. 16

Sociodemographic factors and healthcare-related behaviors may further contribute to this pattern. Although healthcare services are readily accessible in our country, lower educational attainment—more common in older age cohorts—may limit effective use of specialized care. In this context, frequent use of simple analgesics, which constituted the majority of overused medications in our sample (83.7%), may reflect both prescribing practices and patient-related factors. 8 Differences in medication overuse patterns across countries have been attributed to healthcare policies and prescribing habits, which should be considered when interpreting our findings. In our country, opiates and some combined analgesics are in the group of controlled drugs that can only be purchased with a prescription, which we believe reduces their use, given physicians are more selective when prescribing these drugs. Although triptan is not subject to control, we think that it is underutilized because it is not covered by insurance services unless prescribed by a neurologist and it is a relatively expensive drug group.

Another potential contributor to medication overuse may be suboptimal adherence to prophylactic treatments, even when such treatments are prescribed. Reluctance to use antidepressant medications, which are commonly employed in headache prophylaxis, has been reported in clinical practice and may be influenced by concerns about side effects and social stigma. This issue warrants further investigation in future studies.

A further novel finding of this study concerns pain beliefs. Patients with MOH demonstrated significantly stronger organic pain beliefs than those with EH. Previous research has shown that higher organic pain beliefs are associated with greater baseline disability but may also predict better improvement following treatment. 42 In line with the theoretical framework proposed by Edwards et al., the predominance of organic pain beliefs in MOH patients may positively influence adherence to appropriate treatment strategies and coping mechanisms. 20

Pain catastrophizing emerged as a key cognitive feature distinguishing CH from EH. The PCS scores were similarly elevated in MOH and CH groups and significantly higher than in EH. This finding aligns with earlier studies reporting an increased risk of MOH among chronic migraine patients who catastrophize pain. 43 44 45 Together, these results suggest that pain catastrophizing is more closely linked to headache frequency than to subtype and may represent an important cognitive target in management.

Overall, our findings emphasize that MOH should not be viewed solely as a pharmacological consequence of excessive analgesic intake but rather as a condition shaped by specific cognitive and psychological processes. By highlighting pain beliefs and catastrophizing as distinguishing features of MOH, this study contributes novel insights that may inform more individualized and multidimensional approaches to headache management.

### Strengths of the study

This study included both CH and EH groups as controls for the MOH group. This design allowed a clearer comparison of somatization, hypochondriasis, and pain-related cognitive tendencies between individuals who progressed to MOH and those who did not, despite undergoing a similar chronification process.

### Limitations to the study

As a cross-sectional study, causal relationships cannot be inferred, particularly with respect to whether certain personality traits predispose individuals to medication-overuse headache or whether these traits emerge as a consequence of chronic pain over time. The MOH group was significantly older than the EH group; although this condition is more prevalent in the fourth and fifth decades of life, an age-matched design would have reduced any potential confounding effects. Additionally, education levels were unevenly distributed across the study groups and may have influenced pain-related beliefs and coping strategies. In the sociocultural context of our country, lower educational attainment is more commonly observed in older age cohorts; therefore, differences in education may partially reflect age-related cohort effects rather than an independent influence of education. This potential overlap should be considered when interpreting the present findings.

In conclusion, there was a higher frequency of MOH among women with lower education levels, married individuals, and those who were unemployed. Similar to other chronic conditions, depression and anxiety tendencies were higher in this group. The MOH patients also showed stronger organic pain beliefs and greater helplessness, magnification, and rumination compared with the EH group, and demonstrated levels of pain catastrophizing comparable to the CH group.

Interestingly, hypochondriasis scores were lower in the MOH group than in the CH group, suggesting that these patients have less illness-focused worry, despite high levels of pain catastrophizing. This may indicate the presence of a subgroup within MOH characterized by habitual analgesic use rather than illness-focused preoccupation, ultimately leading to CH.

From a clinical perspective, these findings underscore that medication-overuse headache should not be regarded solely as a consequence of excessive analgesic intake, but rather as a condition shaped by distinct cognitive and emotional pain-related processes. Routine clinical assessment of pain catastrophizing, pain beliefs, and affective symptoms may help identify patients at higher risk for persistent overuse and relapse.

Within the biopsychosocial framework, individualized assessment of each patient's personality traits, pain beliefs, and cognitive responses to pain is essential. Therefore, tailored interventions—including cognitive–behavioral therapy, pharmacological treatment, and social support—may improve outcomes. The guiding principle remains: 'There is no disease, only the patient.'
